# Current clinical practices of cytoreductive surgery (CRS) and hyperthermic intraperitoneal chemotherapy (HIPEC)

**DOI:** 10.1515/iss-2023-0055

**Published:** 2024-03-14

**Authors:** Miklos Acs, Maximilian Babucke, Maximilian Jusufi, Zsolt Kaposztas, Przemyslaw Slowik, Matthias Hornung, Hans J. Schlitt, Ivan Panczel, Judit Hevesi, Jonas Herzberg, Tim Strate, Pompiliu Piso

**Affiliations:** Department of General and Visceral Surgery, Hospital Barmherzige Brüder, Regensburg, Germany; Department of Surgery, University Medical Center Regensburg, Regensburg, Germany; Department of General and Visceral Surgery, AK Barmbek, Hamburg, Germany; Department of Surgery, Somogy County Kaposi Mor Teaching Hospital, Kaposvar, Hungary; Faculty of Medicine, Semmelweis University, Budapest, Hungary; University of Regensburg, Regensburg, Germany; Department of Surgery, Krankenhaus Reinbek St. Adolf-Stift, Reinbek, Germany

**Keywords:** cytoreductive surgery, hyperthermic intraperitoneal chemotherapy, peritoneal metastasis, therapeutic indication

## Abstract

Treatment of peritoneal surface malignancies makes physicians face demanding and new-fangled problems, as there are many uncertain aspects considering the outcomes of affected patients’ prognoses. Cytoreductive surgery (CRS) and hyperthermic intraperitoneal chemotherapy (HIPEC) are associated with favorable long-term outcomes in carefully selected patients with peritoneal metastases (PM). We aim to summarize the current results about the initial malignancies and their peritoneal spreads. The current literature has been scrutinized, and studies between 2016 and 2022 were included wherein long-term, progression-free (PFS), and overall survival (OS) data were considered relevant information. Medline, Embase, and Google Scholar have been the main sources. Hereby, we cover all the primer malignancies: gastric, ovarian, and colorectal cancers with peritoneal metastases (PM), malignant peritoneal mesothelioma, and pseudomyxoma peritonei. Examining the advances in the current peer-reviewed literature about the indications of cytoreductive surgery (CRS) and hyperthermic intraperitoneal chemotherapy (HIPEC), target groups, risk factors, and other influencing elements, we intend to provide a complex state-of-the-art report, establishing the relevant aspects of that emerging treatment method.

## Introduction

Solid tumors most frequently metastasizing to the peritoneum include colon carcinoma, gastric carcinoma, mucinous and nonmucinous appendiceal neoplasms, and ovarian carcinoma. In addition, there are the rare peritoneal tumors such as diffuse peritoneal mesothelioma or primary peritoneal carcinoma. Treating these metastatic conditions makes physicians face demanding and new-fangled problems, as there are many uncharted pathways considering the outcomes of affected patients’ prognoses. These peritoneal metastases from different origins were considered incurable and end-staged diseases until the early 1990s, and patients were only offered palliative systemic therapy or supportive care. Since then, a paradigm shift occurred in a manner that these diseases are now considered regional rather than systemic metastases, thus allowing a curative intended approach in selected cases. Consequently, multimodal treatment including cytoreductive surgery (CRS) and hyperthermic intraperitoneal chemotherapy (HIPEC) has emerged, which changed the previous course of the diseases and prolonged survival rates significantly. The outmost aim of CRS is to remove all visible peritoneal metastatic lesion due radical multivisceral surgery in the abdominal cavity, while HIPEC targets the free-floating cancer cells and microscopic metastases of the peritoneum [1]. This new chapter in the treatment of peritoneal surface malignancies is currently evolving, with increasing information and data on individual diseases being obtained. Today, peritoneal metastasis of colorectal (including appendiceal), gastric, ovarian carcinoma, pseudomyxoma peritonei (PMP), and primary malignancies of the peritoneum (malignant peritoneal mesothelioma, primary peritoneal cancer) represent the most frequent indications for multimodal therapy. Hereby, we aim to provide a comprehensive overview of the state of the art in these malignancies. Table 1 shows the general selection criteria for the feasibility of CRS.

**Table 1:** General selection criteria for feasibility of CRS.

General selection criteria
Eastern Cooperative Oncology Group (ECOG-status)<2
Absence of diffuse metastasis to the small intestine and mesentery
Absence of extra-abdominal metastasis (exception in ovarian cancer, cardiophrenic lymph nodes, local pleural carcinomatosis)
Complete surgical cytoreduction appears possible based on preoperative diagnostics and intraoperative assessment
Multimodal therapy at a specialized center

## Colorectal cancer

Colorectal carcinoma (CRC) is one of the most common malignancies worldwide. Epidemiological data show a comparable incidence of synchronous and metachronous peritoneal metastasis (4.3 vs. 4.2 %, respectively) [2]. The prognosis of patients with peritoneal metastatic CRC is poor with a median survival of 7 months [3].

The main risk factors for the development of peritoneal carcinomatosis following an initial curative therapy for CRC were identified as advanced tumor stage (T4 and/or N+ tumors), the presence of a tumor-related perforation at the time of primary surgery, young age, an unfavorable histology (signet ring cell carcinomas, mucinous tumor) and incomplete primary resection [4, 5].

In case of peritoneal colorectal carcinomatosis in selected patients, cytoreductive surgery (CRS) and hyperthermic intraperitoneal chemotherapy (HIPEC) pose a valid therapeutic option. Following the principles of CRS, the complete removal of all macroscopic tumor masses has to be achieved. This can necessitate the parietal peritonectomy and several visceral resections. In contrast to primary malignancies of the peritoneum, only the affected portions of the peritoneum have to be removed in peritoneal metastatic CRC. The extension of the CRS depends on the localization of the peritoneal seedings measured by the peritoneal cancer index (PCI) [6].

To achieve complete cytoreduction in patients with peritoneal metastatic CRC, major surgical procedures are necessary in most cases. Therefore, optimal patient selection is of great importance [7] (Table 2).

**Table 2:** Selection criteria for CRC patients with peritoneal metastasis.

Selection criteria for CRC patients
Eastern Cooperative Oncology Group (ECOG)-status: 0–2
Absence of diffuse metastasis to the small intestine and mesentery
Absence of extra-abdominal metastasis
Limited peritoneal carcinosis (PCI<12–15)
Complete surgical cytoreduction appears possible based on preoperative diagnostics and intraoperative assessment
Easily resectable liver metastases (maximum 3)
Synchronous and metachronous peritoneal metastasis also eligible
(Limited, resectable extra-abdominal metastasis, as an individual decision)

Patients must be in good general health to withstand the major surgical procedure (ECOG status 0–2). In addition, there must be no extra-abdominal metastases and no extensive involvement of the small bowel or its mesentery. The total extent of peritoneal carcinomatosis should be less than a PCI of 12–15. Finally, based on the distribution pattern, a complete macroscopic cytoreduction has to appear possible [8]. The presence of a locally advanced or perforated tumor does not justify the use of a prophylactic HIPEC as was shown in the recent multicentric COLOPEC trial [9]. For latter patients, there is also no need for an aggressive “second-look” procedure to exclude peritoneal carcinomatosis in the follow-up, as the PROPHYLOCHIP trial has shown [10]. Even if the above criteria are met, the local growth pattern of peritoneal carcinomatosis may contraindicate complete cytoreduction. For example, a multilocular involvement of the bowel that would lead to short bowel syndrome or involvement of the hepatic hilum should pose a contraindication.

To adequately assess small bowel involvement, an explorative laparoscopy justified to be part of the preoperative algorithm to exclude those patients who are not eligible for CRS and avoid unnecessary laparotomies [11].

Synchronous liver metastases are only a relative contraindication. The Milan consensus set a maximum of three easily resectable metastases as the upper limit of liver metastasis to be treated via CRS and HIPEC. Recent studies have shown that even major liver resections combined with CRS and HIPEC can be done without a significant increase of surgical risk though. In any case, those procedures should only be performed on well-selected patients with low PCI and low-volume liver disease [12].

The extent of peritoneal carcinomatosis remains one of the decisive criteria for or against surgical cytoreduction. The vast majority of experts today consider a PCI of 15 to be the upper limit for surgical cytoreduction. However, no clear cut-off can be defined based on actual literature alone. For borderline cases, other criteria are very likely to be equally important in determining the indication: tumor biology, tumor behavior under chemotherapy (patients with progression may not be ideal candidates), and extra-abdominal tumor manifestation or serious comorbidities.

The importance of patient selection according to the extent of peritoneal carcinomatosis was demonstrated by Verwaal et al. [13] who compared patients with peritoneal metastatic CRC treated with standard palliative chemotherapy to those treated with CRS and HIPEC. Favorable was demonstrated for a subgroup of patients in the CRS and HIPEC-group (median survival was 12.6 months in the standard therapy arm and 22.3 months in the CRS+HIPEC arm, p=0.032). Mandatory was complete cytoreduction and, most importantly, limited disease, as the patients with high tumor burden showed similar to lower overall survival compared to the conventionally treated group [13].

The significance of systemic therapy in the adjuvant, neoadjuvant, or perioperative setting remains unclear. Possible benefits of neoadjuvant therapy might be a possible reduction in tumor extension, which in turn can lead to a reduction of the extent of surgery. On the other hand, might a systemic therapy result in worsening of the initial situation due to the toxicity of chemotherapy and an increase in the complication rate [14, 15].

There is no consensus on the “right” chemotherapeutic agents for HIPEC regimens, except that according to the PRODIGE-7 trial publication, high-dose oxaliplatin for 30 min has no value and should be replaced by mitomycin C [16, 17].

The PRODIGE-7 trial [16] was a multicenter, randomized, phase 3 trial from 17 French centers published 2020. It was designed to evaluate the specific benefit of HIPEC in combination with CRS in patients with peritoneal metastatic CRC. After central randomization, patients received bidirectional chemotherapy with high-dose oxaliplatin (hyperthermic, intraperitoneal) and intravenous administration of 5-FU and folinic acid. The primary endpoint of the study was overall survival. After a median follow-up of 63.8 months, median survival was 41.7 months (95 % CI 36.2–53.8) in the HIPEC group and 42.2 months in the CRS group (95 % CI 0.63–1.58, p=0.99). The authors concluded that cytoreductive surgery should be the focus of multimodality therapy for peritoneal metastatic CRC [16].

One point of criticism of the PRODIGE-7 study is the very heterogeneous patient population. The study included patients who had a PCI of 20–25. Another point of criticism was the inclusion of patients who had an incomplete cytoreduction, which has also been shown to lead to a worse outcome. The main conclusion of the study was that CRS is an effective therapy that could lead to an extension of survival in patients with low-volume carcinomatosis up to 42 months [18, 19]. The HIPEC performed in this study did not improve survival.

In summary, cytoreductive surgery (with HIPEC) remains a useful option for patients with peritoneal metastatic colorectal cancer. Patients with a low tumor burden (PCI<15) who are treated in an experienced center and in whom complete macroscopic cytoreduction can be achieved benefit most.

## Gastric cancer

Gastric cancer (GC) is the fifth most common cancer worldwide considering both sexes [20]. This aggressive malignancy can give a wide variety of metastases, whereby up to 40 % of GC patients have synchronous and 46 % metachronous peritoneal metastases after curative surgery [21]. Gastric cancer with peritoneal carcinomatosis has poor prognosis, with an average survival of barely 3–6 months if treated with conventional systemic therapy only [21, 22]. To improve survival, CRS and HIPEC have emerged as a therapeutic alternative for selected patients. Most studies analyzing the role of HIPEC are conducted in Asia, the main reason why they have not gained widespread acceptance in the West.

### Therapeutic indication

Several retrospective studies have reported prolonged survival after CRS and HIPEC. In the western part, the French phase III CYTO-CHIP large study compared CRS with CRS+HIPEC in GC patients with peritoneal metastases. The combined therapy group showed a significantly better outcome than those who received surgical cytoreduction alone. Thus, 3- and 5-year survival rates were better in the HIPEC group (26.2 and 19.8 % vs. 10.8 and 6.4 %) [23]. The largest retrospective registry study in Germany was published by Rau et al. identified 215 patients with synchronous peritoneal metastatic gastric cancer individuals who received CRS with HIPEC and divided them into three groups depending on PCI (0–6, 7–15, and 16–39, respectively). Overall survival differed significantly between the three groups and were 18, 12, and 5 months, respectively, with patients who received complete cytoreduction showing a significantly better outcome.

The first phase III randomized trial investigating the efficacy of HIPEC in peritoneal metastatic gastric cancer was published in 2011 by Yang et al. [24]. They enrolled 68 patients and randomized into two equal groups: one received standard therapy with CRS and the other received CRS and additional HIPEC with mitomycin C and cisplatin over 60–90 min. Median overall survival was 6.5 months in the CRS group (95 % CI 4.8–8.2 months) and 11 months in the CRS+HIPEC group (95 % CI 10–11.9 months, respectively). Multivariate analysis identified HIPEC, complete surgical cytoreduction, preoperative chemotherapy, and synchronous metastasis as independent predictors of better oncologic outcome [24]. A two-arm multicenter randomized controlled trial (RCT) (GASTRIPEC-I trial) evaluated the role of HIPEC and published results in 2021 [25]. Patients in the standard arm (CRS) received surgical cytoreduction, and patients in the experimental arm (CRS+HIPEC) also received HIPEC with mitomycin C and cisplatin over 60 min at 42 °C temperature in addition to surgery. All patients were systemically pretreated with EOX. There were no differences in overall survival (OS), which was 14.9 months in the whole population: CRS only 14.9 months (95 % CI 7–19.4), CRS+HIPEC group 14.9 months (95 % CI 8.7–17.7), p=0.1647. PFS was significantly better in the HIPEC group (CRS 3.5 months (95 % CI 3–7), CRS+HIPEC 7.1 months (95 % CI 3.7–10.5), p=0.00472), metastasis free survival (MFS) was also significantly better in the HIPEC group (CRS 9.2 months [95 % CI 6.8–11.5], CRS+HIPEC 10.2 months [95 % CI 7.7–14.7], p=0.0286) [25].

To evaluate the benefit of HIPEC in GC with PM, a large meta-analysis has been conducted from 1985 to 2016 to compare survival after CRS only with CRS+HIPEC. Data showed a benefit in favor of the HIPEC group with a median survival of 11.1 vs. 7.06 months in the control group (p<0.001) [26]. An advantage in the survival rate was found in favor of the HIPEC group at 1-year follow-up (risk ratio RR=0.67, 95 % CI 0.52–0.86), but no statistical difference was found at 2 and 3 years [26].

The results of the controlled two arm Dutch study PERISCOPE II [27] are awaited. This study compares CRS and HIPEC vs. palliative systemic chemotherapy at patients with T3–T4 gastric tumors and limited PM (PCI<7).

Patient selection is crucial for achieving maximum survival benefit as it has been shown by an international group study [28] (Table 3). Complete macroscopic cytoreduction is of essential importance for overall survival of patients with peritoneal metastatic gastric carcinoma. This meta-analysis confirmed that the patients in whom a CCR-0 or CCR-1 (no macroscopic tumor remnants or remnants<2.5 mm) situation could be achieved showed significantly better survival at 1, 3, and 5 years than those in whom no cytoreduction was achieved [28]. Herein, in the feasibility to achieve complete cytoreduction, the tumor burden (measured by the peritoneal cancer index PCI) plays a decisive role. Several studies report the association of low PCI with improved survival; nevertheless, no exact PCI cut-off has yet been determined [21]. A PCI cut-off of seven are supported by several authors [21, 29, 30].

**Table 3:** Selection criteria for peritoneal metastatic gastric cancer.

Selection criteria for peritoneal metastatic gastric cancer
Eastern Cooperative Oncology Group (ECOG)-status: 0–2
Absence of diffuse metastasis to the small intestine and mesentery
Absence of extra-abdominal metastasis
Limited peritoneal carcinosis (PCI<6, maximum 9)
Complete surgical cytoreduction appears possible based on preoperative diagnostics and intraoperative assessment
No exclusion criteria during diagnostic laparoscopy
Synchronous peritoneal metastasis
Good response after inductive chemotherapy

According to the Chicago consensus document, patients with peritoneal metastases from gastric cancer should be considered and treated in the framework of clinical trials [31].

### Prophylactic indication

Currently, investigators try to establish effective preventive methods for patients at high risk for peritoneal carcinomatosis after R0 Gastrectomy. Since PM develops in 46 % of patients after curative intent gastrectomy [21], an enrolling international phase III trial (PREVENT; NCT04447352) was designed to compare conventional therapy (gastrectomy) with CRS and HIPEC in locally advanced adenocarcinoma patients (T3–T4, N0, M0) after FLOT (docetaxel, oxaliplatin, leucovorin, and 5-fluorouracil) neoadjuvant chemotherapy in both groups.

In a further GASTRICHIP trial, the conductors try to explore the prophylactic aspects of HIPEC, and their results are expected in 2024 [32]. GASTRICHIP is an approaching, open-label multicentric randomized phase III clinical study focusing on HIPEC with oxaliplatin applied on gastric cancer patients with serosa or lymph node involvement with positive cytology at peritoneal washing, combined with perioperative systemic chemotherapy and D1–D2 curative gastrectomy. Patients were treated due gastrectomy with or without HIPEC. According to the investigators, it is highly indicated that HIPEC will take up an even more important role as a prophylactic rather than a therapeutic setting [32].

## Malignant peritoneal mesothelioma (MPM)

Malignant peritoneal mesothelioma (MPM) is the second most common mesothelial cell tumor after malignant pleural mesothelioma [33]. MPM is a rare primary tumor arising from the mesothelial cells lining the peritoneal cavity. Its incidence rate in industrialized countries is reported to be 0.5–3 per 1,000,000 in men and 0.2–2 per 1,000,000 in women [34]. From all mesotheliomas diagnosed, only 5–20 % are originating from the peritoneal surface [35, 36]. Without treatment, the median survival is sparse and counting 6–12 months. The diagnosis of a MPM is difficult in the absence of pathognomonic symptoms. Usually, the identification of these patients is through clinical abnormalities of an abdominal circumferential mass (ascites, tumor mass, and pain), patients with B-symptoms combined with signs of an inflammatory bowel disease, and patients with acute problems requiring surgery. There are no recommendations for screening measures related to MPM.

Historically, the treatment of MPM was confined to systemic chemotherapy, palliative surgery, and abdominal radiotherapy [37]. These types of treatments had plenty of side effects and median survival was less than a year [38]. Untreated, the aggressive growth of the MPM lead to rapid progression, significant morbidity, and ultimately death. When the concept of aggressive surgical cytoreduction and hyperthermic intraperitoneal chemotherapy was introduced in the mid of 1990s as a treatment option for the MPM by Sugarbaker [39], it made long-term outcomes possible. Because of the rarity of this disease, there are still no randomized trials conducted. That’s why it is even more important to gain data from all available sources. Nevertheless, CRS+HIPEC has become the mainstay of the therapy for selected MPM patients [40].

In the last decade, several retrospective register studies were published to evaluate feasibility and long-term survivals after multimodal therapy. Data from 566 patients with a MPM diagnosis from 1993 to 2016 from the Dutch Cancer Registry [41] showed that overall survival increased over time and surgery as a treatment became more common in recent years. With multivariable analysis, better survival was independently associated with surgery (HR, 0.33; 95 % CI, 0.23–0.48), HIPEC (HR, 0.33; 95 % CI, 0.21–0.55), and systemic chemotherapy (HR, 0.61; 95 % CI, 0.49–0.76). Additional factors for a longer survival were female sex (HR, 0.65; 95 % CI, 0.53–0.81) and an age between 65 and 74 (HR, 1.55; 95 % CI, 1.25–1.92) than an age older than 75 years (HR, 2.00; 95 % CI, 1.59–2.51). Significantly worse outcomes were associated with a sarcomatoid or biphasic subtype when compared with the epitheloid type [41].

In another register study from the US American National Cancer Data Base [42], similar results were gathered. Data from 1,514 patients were selected, 379 (25 %) underwent observation, 370 (24 %) received chemotherapy only, 197 (13 %) CRS alone, 352 (23 %) CRS/chemo, and 216 (14 %) CRS/HIPEC. Median overall survival in the corresponding groups were 6, 17, 21, 52, and 61 months (p<0.001). The two-year survival rates with CRS/HIPEC (50 %) were similar to the CRS/chemo group (44 %, HR for death 1.115, 95 % CI 0.867–1.435). However, CRS/HIPEC was noticeably superior compared with CRS alone (22 %, HR for death 1.859, 95 % CI 1.378–2.509), chemotherapy alone (18 %, HR for death 1.843, 95 % CI 1.450–2.341), and the observation group (8 %, HR for death 2.903, 95 % CI 2.270–3.702) [42].

As shown prognosis for MPM patients can broadly differ, it is important to classify patients. Yan et al. [43] proposed a TNM staging method where the T stage was categorized by quartiles of the PCI: T1 (PCI 1–10), T2 (PCI 11–20), T3 (PCI 21–30), and T4 (PCI 30–39). Survival of MPM patients with positive lymph nodes (N1) and extra-abdominal metastases (M1) are similarly poor resulting in three stages. Stage I (T1N0M0), stage II (T2N0M0 or T3N0M0), and stage III (T4, N1 and/or M1 disease) demonstrated a 5-year OS survival of 87, 53, and 29 % respectively. Other relevant factors for worse survival are male sex, advanced age (>60 years), high-grade histology (biphasic, sarcomatoid), unfavorable disease distribution for complete cytoreduction, and, as shown recently by Li et al.[44], preoperative thrombocytosis. Elevated preoperative platelet count>367.000/mm^3^ was significantly associated with poorer overall survival. Median survival was 13 months for patients with thrombocytosis and 58 months for normal platelet count patients (p<0.001). Beneficial factors are good performance status (ECOG 0 or 1), disease distribution amenable for complete or near-complete (CCR0 or CCR1) cytoreduction, age<60 years, female sex, epithelioid histology, and a low PCI [45] (Tables 4 and 5).

**Table 4:** Favorable and unfavorable factors regarding patient survival and outcomes with MPM.

	Favorable factors	Unfavorable factors
Performance status	ECOG 0–1	≥2
Age	≤60 years	>60 years
Sex	Female	Male
Histology	Epitheloid	Sarcomatoid, biphasic
Ki67 proliferation index	≤9 %	>9 %
Platelet count	≤367.000/mm^3^	>367.000/mm^3^
PCI	<17	≥17
Disease distribution	CCR0 – CCR1 possible	CCR2

These factors are not absolute contraindications but should help in decision-making for finding suitable patients for cytoreductive surgery.

**Table 5:** Histologic classification of pseudomyxoma peritonei according to PSOGI [73].

Pathologic lesion	Criteria
Acellular mucin	– Mucin within the peritoneal cavity without neoplastic epithelial cells
Low-grade mucinous carcinoma peritonei (DPAM)	– Epithelial component typically scanty – Minimal cytologic atypia – Strips, gland-like structures, or small cell clusters
High-grade mucinous carcinoma peritonei (PMCA)	– Relatively more cellular – Cribriform growth pattern – High-grade cytologic atypia – Numerous mitoses
High-grade mucinous carcinoma peritonei with signet rings cells (PMCA-S)	– Any lesion with a signet ring cell component, that is, round cells with intracytoplasmic mucin pushing the nucleus against the cell membrane

DPAM, disseminated peritoneal adenomucinosis; PMCA, peritoneal mucinous carcinomatosis; PMCA-S, peritoneal mucinous carcinomatosis with signet ring cells.

**Figures 1–4:**
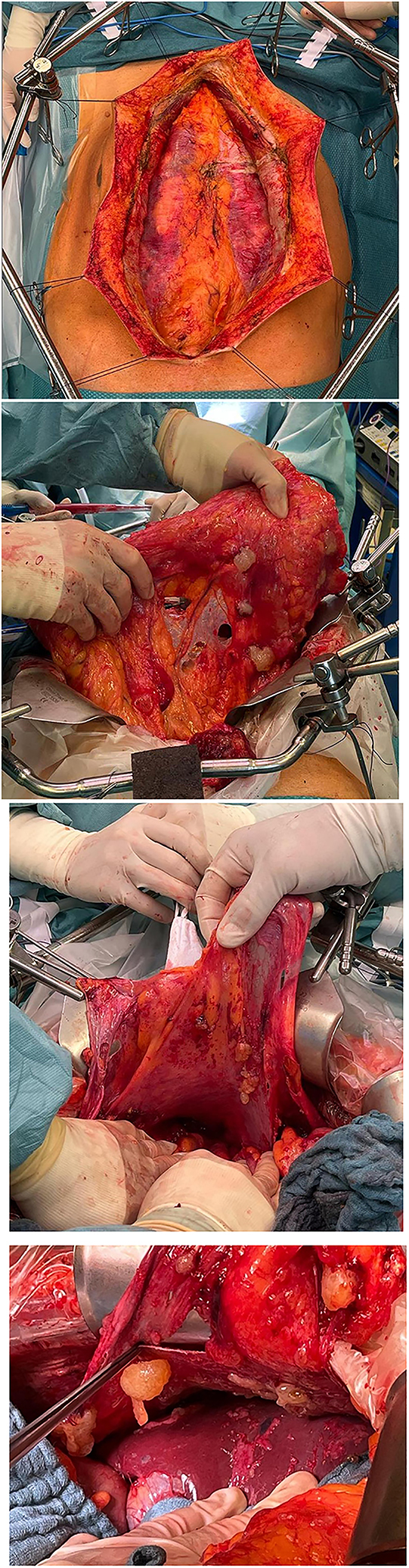
These images were taken during cytoreductive surgery of a MPM patient with an epitheloid subtype. Shown is the tumor distribution along the parietal and visceral peritoneum (2. mesenterium, 3. parietal peritoneum, 4. liver surface). Picture 1 shows the extraperitoneal approach of a complete parietal peritonectomy.

In another retrospective series of 249 patients receiving CRS and HIPEC from the RENAPE database, it was shown that when comparing two drugs vs. one drug as the HIPEC agents, the overall survival for patients with a CC-0 resection and epitheloid subtype (HR 0.25, 95 % CI 0.09–0.72; p=0.009) was significantly longer. Even more the PFS improved as well [46]. The study could not answer which HIPEC regime should be favored, because no statistical difference for overall survival was found when comparing cisplatin + mitomycin c vs. cisplatin + doxorubicin (HR 1.00, 95 % CI 0.99–2.47, p=0.99) or cisplatin + doxorubicin vs. oxaliplatin + irinotecan (HR 0.52, 95 % CI 0.17–1.60, p=0.25). A platinum-based HIPEC consisting of at least two agents should be favored [46]. While surgery has its proven central role in the multimodal therapy, the significance of HIPEC remains unclear. For this purpose, prospective randomized controlled studies are necessary (Figures 1–4).

Kusamura et al. figured that the Ki67 proliferation index along with the PCI is a good prognostic tool to predict OS for the MPM. When Ki67 proliferation index>9 % and a high PCI>17 is evaluated, patients are unlikely to benefit from CRS+HIPEC. These patients may better be treated with systemic chemotherapy first and be reevaluated after 2–3 cycles [45]. Nevertheless, another study with data from the French RENAPE network with comparable clinicopathological and prognostic factors regarding the MPM suggests a potential benefit of adjuvant chemotherapy with an improvement in 5-year overall survival to 67 % from 56 % without systemic therapy [47].

There are no data for a specific regimen for a follow-up, but after complete cytoreduction, close 3-month follow-up with imaging in the same modality (preferably computer tomography) as preoperatively is recommended above all. Furthermore, radiologists could use the “PAUSE” (peritoneal cancer index, ascites and abdominal wall disease, unfavorable sites of involvement, small bowel and mesenteric disease, and extraperitoneal disease) technique, which helps in surgical decision-making for MPM patients [48].

The quality of the resection depends strongly on the skills of the surgeon and in addition the radicality (CCR-0 or CCR-1 is regarded as complete resection) is highly important for the resulting prognosis [42]. Furthermore, CRS+HIPEC has a high morbidity and mortality, but when executed in specialized centers, it is often safer than other similar-risk oncological procedures [49].

In conclusion, the MPM is a rare disease and tough to diagnose. Clinicians should initiate further diagnostic in cases of unclear ascites or peritoneal carcinomatosis from unknown origin and refer these patients to a specialized center for further therapy since several patients may greatly benefit from cytoreductive surgery and HIPEC.

## Ovarian cancer

In ovarian, fallopian tube and primary peritoneal carcinomatosis, peritoneal metastases belong to the natural history of the primary and recurrent disease. The fact that 75 % of cases are diagnosed first in advanced stages [50] (Fédération Internationale de Gynécologie et d’Obstétrique FIGO stage III–IV) calls for potential curative options. The cornerstone of the treatment is CRS followed by adjuvant platinum-based chemotherapy and biological treatments [51]. More than 50 clinical trials examined the use of HIPEC for treating ovarian cancer within the last 2 years [52]. Yet national and international guidelines have changed barely with the extension of protocol with HIPEC, whereby the National Comprehensive Cancer Network (NCCN) 2019 guideline [51] and the French clinical practice guideline [53] consider the addition of this treatment after interval debulking surgery.

### Role of HIPEC in primary ovarian cancer

In primary ovarian cancer to perform CRS and HIPEC, there are two possible therapeutic scenarios: primary CRS with HIPEC after the diagnosis of the disease or neoadjuvant chemotherapy with 3–6 cycles of carboplatin + paclitaxel followed by interval CRS and HIPEC.

To date, four randomized controlled trials have been published with inclusion of 519 patients (259 in intervention and 260 in control group) to the investigational use of HIPEC in primary ovarian cancer [54], [55], [56], [57]. Two of these studies examined interval CRS+HIPEC after neoadjuvant chemotherapy (NACT) [55, 56], and in the study by Lim et al. [54] at 42 % of the randomized patients preceded chemotherapy prior CRS with or without HIPEC. Four years ago, the research group of van Driel et al. published their first ever phase 3 randomized controlled trial in epithelial ovarian cancer showing the potential benefit of neoadjuvant chemotherapy accompanied by interval cytoreductive surgery and HIPEC [56]. They have shown an increase in recurrence-free survival of 10.7–14.2 months and median overall survival from 33.9 to 45.7 months in the conventional (CRS only) and experimental arm (CRS+HIPEC), respectively [56]. In accordance with these finding, Cascales-Campos et al. [55] reported better DFS and OS with CRS and HIPEC after NACT. In the Korean randomized trial [54], the addition of HIPEC did not improve PFS and OS in patients with advanced epithelial ovarian cancer (p=0.43 and p=0.52, respectively). Although in a subgroup analysis, the addition of HIPEC to interval cytoreductive surgery provided an improvement of progression-free and overall survival (PFS 15.4 months in the control group and 17.4 months in the HIPEC group p=0.04, and OS 48.2 months in the control group and 61.8 months in the HIPEC group, respectively, p=0.04). Furthermore, according to the preliminary results of Diaz-Montes et al. [57] after enrollment of 19 patients so far, an apparent advantage of CRS+HIPEC in terms of disease-free survival (DFS) and overall survival at 2 years could be observed (69 % for the CRS+HIPEC group and 59 % for the control group (p=0.75) and 89 % for the CRS+HIPEC group and 77 % for the control group (p=0.75), respectively) [57].

In conclusion, after reviewing these findings and in line with other investigators [58], the firm conclusion can be driven that there is sufficient evidence for the superiority of CRS+HIPEC in the interval setting in the treatment of epithelial ovarian cancer. To date, there is no published phase III trial on the impact of HIPEC in the upfront setting. The OVHIPEC-2 study started in 2019 by van Driel aims to roll up the additional effect of HIPEC after CRS with CCR-0 or CCR-1 resection in primary setting in FIGO stage III patients [59].

### Role of HIPEC in recurrent ovarian cancer

Recent findings in DESKTOP III confirmed the part of well-placed cytoreduction in the treatment of recurrent ovarian cancer [60]. It was affirmed by another crucial observation that the result of HIPEC is related to PCI; thus, the chance to reach optimal cytoreduction significantly increased. This treatment should be emphasized at experienced centers and used by well-trained multidisciplinary groups following a targeted and precise selection of patients [61].

To date, two randomized controlled trials have been published with inclusion of 218 patients (109 in the HIPEC and 109 in the control group) to the experimental use of HIPEC in recurrent ovarian cancer [62, 63]. The first ever randomized clinical trial in recurrent ovarian cancer was reported by the Greek research group [63] in 2015 demonstrating a significant benefit in mean OS for CRS+HIPEC followed by systematic chemotherapy vs. CRS with systematic chemotherapy alone (26.7 vs. 13.4 months, respectively, p<0.006). On the contrary, Zivanovic et al. [62] randomized 98 patients to carboplatin HIPEC or no HIPEC and found no significant difference in median overall survival (52.5 vs. 59.7 months, respectively; hazard ratio HR=1.39; 95 % CI 0.73–2.67; p=0.31). Thus, the impact of HIPEC in recurrent ovarian cancer remains inconsistent at present, and further results from randomized trials are eagerly awaited.

### Pseudomyxoma peritonei (PMP)

Pseudomyxoma peritonei (PMP) is one of the rarest peritoneal carcinomatosis and refers to a clinical syndrome, which is characterized by abundant production of extracellular mucin in the peritoneal cavity. In the vast majority of cases, it originates from the appendix, but other neoplastic and non-neoplastic diseases of other primary localization (ovary, small and large intestine, urachus, pancreas, bile ducts, stomach, kidney and others) are also with less than 10 % of the cases rather rarely the cause of a PMP [64]. It is estimated that in 2 out of 10,000 laparotomies, the surgeon is unexpectedly confronted with the clinical picture of a PMP [65]. The disease exhibits only slow progression, a low tendency to hematogenous or lymphogenous metastasis, and infiltrative growth compared to typical peritoneal carcinomatosis from other origins. The very low incidence of 0.2–2 cases per million inhabitants [66] explains the cause of absence of randomized controlled trials. All data so far are based on single and multi-institutional studies, many of which before the unification of developed histologic classification system.

The terminology and classification were inconsistent for a long time; thus, in 2016, a unification of the nomenclature and classification of epithelial appendiceal tumors and PMP was introduced by the Peritoneal Surface Oncology Group International (PSOGI) led by Carr et al. [67] in a consensus conference. Other significant and widely used classification systems published in 2017 were the AJCC staging system (8th edition, TNM) and in 2019 the WHO classification (5th edition) [68, 69]. After the uniform introduction and acceptance of the nomenclature, it is only possible to compare the data and studies among. It is important to emphasize that both the appendiceal neoplasia (primarius) and the produced extracellular mucin are separately examined histologically by the pathologist, since the prognosis depends essentially on differentiation and cellular content of epithelial cells in the extraluminal mucin (Table 1). Another crucial finding in this regard that in 3.7–11.7 % of the cases, a discordant histological diagnosis between primarius and peritoneal manifestation is found [70, 71]. Moreover, it is pivotal to differentiate the intraperitoneal mucinous spread originating from low- or high-grade mucinous neoplasms (LAMN and HAMN) of the mucinous adenocarcinoma of the appendix because of the substantial difference in these entities [72].

With pseudomyxoma peritonei associated appendiceal neoplasms:

– Low-grade appendiceal mucinous neoplasm (LAMN)
– High-grade appendiceal mucinous neoplasm (HAMN)
– Mucinous adenocarcinoma (well-, moderately, poorly differentiated)
– Poorly differentiated mucinous adenocarcinoma with signet ring cells
– Signet ring carcinoma

Despite the very heterogeneous tumor biology and the varying malignant potential, the clinical manifestation of PMP is relatively uniform. The thoroughly accepted standard of therapy in disseminated PMP is the combination of CRS and HIPEC [74, 75]. The classical tumor distribution confined to the peritoneal cavity and – despite frequently occurring high tumor load – the often noninvasive or less invasive tumor growth allows the performance of curative intent surgery for 2/3 of patients despite high tumor burden [76].

Completeness of cytoreduction (CCR-0: complete cytoreduction, CCR-1 leaving<2.5 mm of disease behind) is a significant prognostic indicator for improved survival [77, 78]. Chua et al. evaluated the long-term outcome at 2,298 patients who underwent CRS+HIPEC and found that incomplete cytoreduction was associated with worse PFS and OS. Five-year overall survival was 24 % after incomplete cytoreduction compared with 85 and 80 % after CCR-0 and CCR-1 resection, respectively [78]. In another study by Chua et al. [79] at 106 patients of whom 76 % received HIPEC accompanied with CRS optimal cytoreduction (CCR-0 or CCR-1) was achieved in 91 % of the cases. In none of the both studies by Chua et al. [78, 79] was HIPEC (mitomycin in most cases) independently associated with overall survival (p>0.05). Nevertheless, HIPEC was independently associated with progression-free survival (p=0.030) [78].

**Figure 5:**
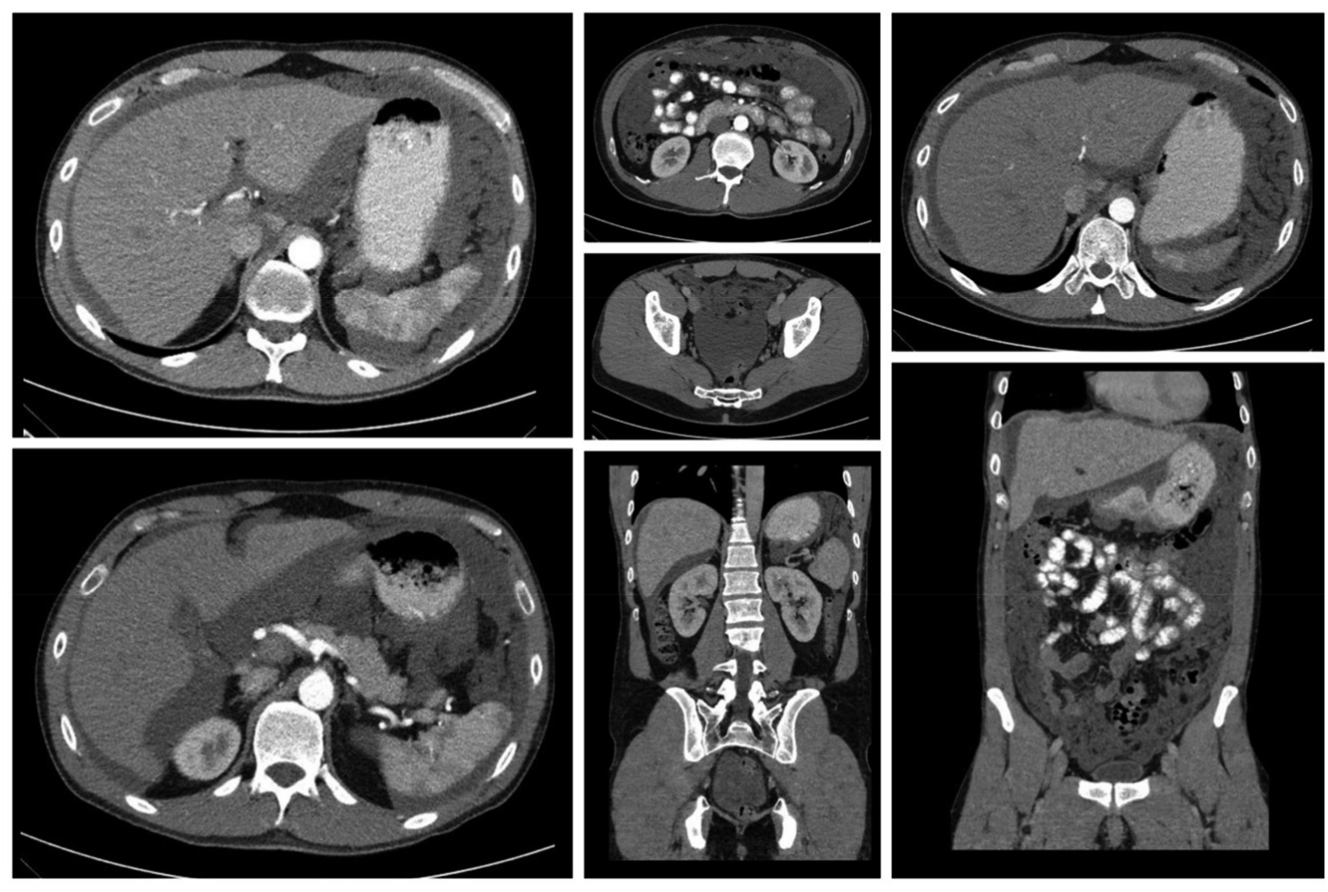
CT images of a patient with extensive PMP tumor burden.

In a recent French study, Benhaim et al. [80] published a series of 68 CRS+HIPEC procedures in patients with extensive tumor burden, which was defined as a peritoneal cancer index (PCI)≥28. The disease-free survival was 45 % in the extensive PMP and 78 % in the nonextensive PMP group (p<0.0001). The 5-year overall survival rates were 70 and 90 % in the extensive PMP and nonextensive PMP group, respectively (p<0.021) [80]. Figure 5. CT images show a 40-year male patient with pseudomyxoma peritonei originating from low-grade appendiceal mucinous neoplasm (LAMN) with extensive tumor involvement (PCI 36), undergoing subtotal gastrectomy with Omega loop reconstruction, colectomy, omentectomy, rectum resection and reconstruction due ileorectostomy, cholecystectomy, splenectomy, complete peritonectomy, liver capsule resection, peritonectomy of the small bowel mesentery with CCR-1 resection followed by HIPEC with mitomycin for 90 min.

To avoid significantly increased morbidity from extensive cytoreduction due to high tumor burden, recently two researcher groups tried to solve the problem of high major morbidity by a so-called “two-stage CRS/HIPEC” [81, 82]. Hereby is an interval of approximately 4 months between the two procedures used for patient recovery. With this new strategy, severe complications could be reduced and were reported in only 12.5 and 25 % of the patients, respectively [81, 82]. Furthermore, complete macroscopic cytoreduction was achieved in 88 and 100 % after the second procedure, respectively, and no mortality was reported. In the French study by Sgarbura et al., all patients were tumor-free after a median follow-up of 31.5 months. Nevertheless, this approach can be maintained just in a case low biological aggressiveness (acellular tumors, low-grade PMP) due to the long interval between surgeries. However, in about 20 % of patients, a complete cytoreduction is not feasible due to extensive abundant tumor masses [77, 83]. For these individuals repeated debulking operations is an option to reduce abdominal distension by continuous tumor progression. With this symptom control approach, in a meta-analysis across five studies and 766 patients achieved a 5-year survival rate of 18.5 % for patients with high-grade tumors and 45.2 % for low-grade tumors [84].

### Special comment on HIPEC

Prerequisites for maximum utilization of the therapeutic effect of multimodal therapy are the absence of extra-abdominal metastases, limited peritoneal metastasis (measured by PCI) depending on tumor entity, and macroscopically complete tumor removal. However, PCI value alone is not the only decision criterion in assessing patients’ suitability for CRS and HIPEC, other patient-related factors such as age, comorbidities, histopathologic, and molecular pathologic features of the tumor should be considered before deciding on CRS with HIPEC [85]. In this context, laparoscopy is quite well suited to exclude tumor dissemination into irresectable structures, thus selecting patients who will not benefit from burdensome therapy [11]. After reviewing the literature, we come to the conclusion that surgery carries the outmost importance of therapy, whereas the impact of HIPEC remains controversial. Nevertheless, numerous randomized controlled trials (RCT) with both therapeutic and prophylactic indication are ongoing or planned (13 RCTs in colorectal cancer, 16 RCTs in gastric cancer, 17 RCTs in ovarian cancer [86]) to evaluate the additional effect of HIPEC.

The main criticism against HIPEC used to be the potentially high rates of complications and potential risk for death and adverse long-term consequences [13, 87]. In a large retrospective study including 2,149 consecutive patients, major grade 3 and 4 complications according to Clavien–Dindo classification occurred in 19.3 % of all patients, and overall, 30-day postoperative hospital mortality was 2.3 % (95 % CI 1.02–3.85) [88]. In another study reporting 25-year experience with 1,125 HIPEC procedures, the grade≥3 morbidity rate was 9.6 % and 30-day mortality rate of 1.5 % [89]. In addition, a most recent review in ovarian cancer emphasized the safety of HIPEC procedure and indicated at level I evidence that major morbidity and risk of mortality is similar in comparison with cytoreduction without HIPEC [58].

One of the main bottlenecks of HIPEC research and consequently in the interpretation and comparison of the result is the remarkable heterogeneity and variety of HIPEC parameters (open vs. closed techniques, dosage of chemotherapeutics, timing of therapy and duration) among centers worldwide [90]. However, there is growing evidence that prolonged exposure time in the peritoneal cavity is not associated with increased morbidity and may contribute prolong survival [90, 91].

## Conclusions

Our understanding is increasingly evolving about the heterogeneous behavior, tumor biology, and therapeutic strategies of peritoneal surface malignancies. However, there are many unanswered and still open questions about the proper use, timing of cytoreductive surgery, and hyperthermic intraperitoneal chemotherapy in certain tumor types. Proven evidence shows that complete macroscopic cytoreduction is of paramount importance for survival in all tumor entities. To ensure this, a thorough patient selection and proper surgical training is a prerequisite.

## Supplementary Material

Supplementary Material
