# Supplementary material for: Current clinical practices of cytoreductive surgery (CRS) and hyperthermic intraperitoneal chemotherapy (HIPEC)
Source: Innov Surg Sci. 2024 Mar 14;9(1):3–15. doi: 10.1515/iss-2023-0055 (PMC11138857; doi:10.1515/iss-2023-0055)
Supplement: Supplementary file 1 — Supplementary Material [file j_iss-2023-0055_suppl_001.pdf]

Reviewer Assessment

P. Piso et al.: Current clinical Practices of Cytoreductive Surgery (CRS) and Hyperthermic Intraperitoneal Chemotherapy (HIPEC)

Reviewers' Comments to Original Submission

Reviewer 1: anonymous

Date received: 13-Dec-2023
Reviewer recommendation: **Accept in present form**
Reviewer overall scoring: **Excellent**

Comments to author:
Excellent review that covers all aspects of this treatment option for peritoneal carcinomatosis. During the production process a check of the use of the English language is highly recommended.
Also please check figures 1-4 - where should they be placed in the manuscript exactly?

Reviewer 2: Seitz, Guido

Date received: 19-Dec-2023
Reviewer recommendation: **Accept in present form**
Reviewer overall scoring: **Excellent**

Comments to author:
Well written. Pages for citation 1 are missing. Please add
